# TLR3 Knockdown Attenuates Pressure‐Induced Neuronal Damage In Vitro

**DOI:** 10.1111/jcmm.70276

**Published:** 2024-12-13

**Authors:** Li Lin, Zhongzhong Lv, Chao Zhou, Taiyang Zhu, Yuting Hu, Xiaoyu Sun, Hui Zhou, Miao Wang, Yongtao Lin, Guoqing Gu, Shang Wang, Yan Zhou, Jingjing Han, Guoliang Jin, Fang Hua

**Affiliations:** ^1^ Department of Neurology Xuzhou Medical University Xuzhou China; ^2^ Department of Neurology Benq Hospital Affiliated to Nanjing Medical University Nanjing China; ^3^ Department of Neurosurgery Benq Hospital Affiliated to Nanjing Medical University Nanjing China; ^4^ Department of Neurology, Nanjing Drum Tower Hospital, Affiliated Hospital of Medical School Nanjing University Nanjing China; ^5^ Xuzhou Medical University Xuzhou China; ^6^ Department of Interdisciplinary Health Sciences College of Allied Health Sciences, Augusta University Augusta Georgia USA

**Keywords:** apoptosis, autophagy, microtubule‐associated protein‐2, mitochondria, pressure‐injured, spinal cord injury, TLR3

## Abstract

The disruption of nerve parenchyma and axonal networks triggered by spinal cord injury (SCI) can initiate a cascade of events associated with secondary injury. Toll‐like receptors play a critical role in initiating and regulating immune‐inflammatory responses following SCI; however, the precise involvement of Toll‐like receptor‐3 (TLR3) in secondary neuronal injury remains incompletely understood. To investigate the potential contribution of TLR3 in mediating neuronal pressure‐induced damage, we established a stress‐induced neuronal damage model using rat anterior horn motor neuron line (VSC4.1), which was subjected to varying levels and durations of sustained pressure. Our findings suggest that pressure induces neuronal damage and apoptosis, and reduced proliferation rates in VSC4.1 cells. Furthermore, this pressure‐induced neuronal injury is accompanied by upregulation of TLR3 expression and activation of downstream TLR3 signalling molecules. Knockdown experiments targeting TLR3 significantly alleviate pressure‐induced motor neuron injury and apoptosis within the anterior horn region while promoting mitochondria‐related autophagy and reducing mitochondrial dysfunction via the TLR3/IRF3 and TLR3/NF‐κB pathways.

## 1 Introduction

Spinal cord injury (SCI) commonly occurs when the spinal cord is continually compressed due to trauma‐induced fractures and vertebral dislocation. Primary injury may result in the destruction of neural parenchyma, disruption of axonal networks and activation of a cascade of events associated with secondary injury [1, 2, 3, 4]. Cellular and molecular responses triggered by spinal cord compression cause prolonged secondary damage, with implications for anterior horn motor neurons [5, 6, 7]. Previous studies have indicated that immune responses to SCI can have either beneficial or detrimental effects on nerve damage and neuroprotection [8]. These effects must be selectively modulated to achieve optimal remediation potential while minimising harmful consequences [9, 10, 11]. It is widely believed that excessive activation of innate immunity is detrimental to neural tissues [12, 13, 14], suggesting that specific targeting of innate immune pathways might provide an approach for improving the outcome of SCI secondary injury.

The Toll‐like receptor (TLR) superfamily of type I transmembrane receptors plays a crucial role in initiating and regulating immune‐inflammatory responses. Upon activation by their ligands, TLRs recruit signalling mediators (mainly MyD88 and TRIF) and downstream protein kinases, resulting in the activation of transcription factor nuclear factor‐kappa B (NF‐κB) and interferon regulatory factor (IRF), which ultimately regulate inflammatory immune responses [11, 15]. Toll‐like receptor 3 (TLR3) plays a crucial role in the innate immune system, making it a prime target for immunomodulation [11, 15]. However, the involvement of TLR3 in SCI remains to be explored.

To investigate the mechanism, pathology and treatment of SCI, a variety of in vivo animal models and in vitro SCI models have been established [16, 17, 18, 19, 20, 21]. However, an in vitro model of SCI that recapitulates the injury caused by compression has not yet been established. In the present study, we used a thermostatic incubator combined with a pressure gas tank and VSC4.1 cells to generate an in vitro model of pressure‐induced cell damage. The effects of modulation of TLR3 on pressure‐injured neurons were evaluated, along with its underlying mechanisms.

## 2 Materials and Methods

### 2.1 Cell Culture

The rat spinal cord anterior horn motor neuron line VSC4.1 (Shanghai Liemai C453) was cultured in Dulbecco's Modified Eagle Medium (DMEM) and supplemented with 10% FBS and 1% penicillin–streptomycin solution. The P2 generation of VSC4.1 was frozen, and cells from the P3–P9 generations were used in subsequent experiments. Cell lines from the P3/P6/P9 generations were authenticated through short tandem repeat analysis, and cell slides were imaged using a light microscope.

For TLR3‐siRNA, a complementary strand of the single‐stranded target gene fragment was synthesised. Plasmid FV‐023 was cleaved using AgeI and EcoRI enzymes, and the resulting large fragments were recovered through 1% agarose gel electrophoresis. Subsequently, the annealed fragment was ligated to the linearised plasmid FV‐023 vector. After transformation and identification, the appropriate plasmid was selected for sequencing, followed by large‐scale extraction of plasmid FV‐023‐rat‐TLR3‐shRNA.

For TLR3 overexpression, a DNA fragment corresponding to the 2718 bp rat TLR3 cDNA sequence was synthesised using the pDONR Vector System (Invitrogen Life Technologies) and then was ligated into NotI/BamHI sites of pcDNA3.1‐Hygro (+) vector. The resulting product was transformed into bacteria, and the correct bacterial colony was selected for verification by PCR. Finally, the large‐scale plasmid pcDNA3.1‐hygro (+)‐rat‐TLR3 was extracted (Figure S1).

### 2.2 Experimental Reagents

Rabbit polyclonal anti‐cytochrome C (15KD) (Wuhan Sanying Biotechnology Co. LTD., 10993‐1‐AP); rabbit polyclonal anti‐P‐P65 (65KD) (Affinity, AF3391); rabbit monoclonal anti‐P65 (65KD) (Cell Signaling, 8242); murine monoclonal anti‐LC3 (14/16KD) (CST, 83506); Cytochrome b5 polyclonal antibody (15KD) (Immunoway, YT1257); murine monoclonal anti‐Caspase3 (35/17KD) (Wuhan Sanying Biotechnology Co. LTD., 66470‐2‐ig); MAP2 polyclonal antibody (85KD) (Wuhan Sanying Biotechnology Co. LTD., 17490‐1‐AP); MAP2 (Phospho‐Thr1616) polyclonal antibody (280kD) (ImmunowayYP1233); rabbit monoclonal anti‐phospho‐IRF‐3 (Ser396) (D6O1M) (45‐55KD) (CST, 29047); rabbit polyclonal anti‐IRF3 antibody (47KD) (Abcam, ab238521); and murine monoclonal anti‐tlr3 (66kd) (Novus, Ddx0470p) were used with horseradish peroxidase (HRP) and sheep or rabbit secondary antibodies (Wuhan BOSTER Biological Technology Co. Ltd., BA1054). Additional reagents included fluorescent (Cy3) labelled sheep anti‐rabbit IgG (Wuhan BOSTER Biological Technology Co. Ltd., BA1031); fluorescent (FITC)‐labelled sheep anti‐rabbit IgG (Wuhan Bode Bioengineering Co. LTD. BA1105); PDONRTM 223‐rat‐TLR3 Cell Counting Kit‐8 (MCE HY‐K0301 59920); LBAD‐mcherry‐EGFP‐LC3 (HB‐AP‐2100001 20070611‐30); TLR3‐rat‐434 (Shanghai, Genepharma Technology Co. LTD, 6927), TLR3‐rat‐655 (Shanghai, Genepharma Technology Co. LTD, 6928), TLR3‐rat‐2867 (Shanghai, Genepharma Technology Co. LTD, 6929); Taq Plus DNA Polymerase (TIANGEN, ET105‐02‐01); T4 DNA Ligase (TRANS, 20325); BamH I (TAKARA, 1010A); Not I (TAKARA, 1166A); DL15000 DNA Maker (TIANGEN, MD110); DL2000 DNA Maker (TIANGEN, MD114); PDONR 223‐Rat‐TLR3 (Kingsrui Biotechnology Co. LTD); the thin agarose gel DNA recovery kit (GENERAY Biotechnology, GK2043‐200); plasmid preparation kits (GENERAY Biotechnology, GK2004‐200); gold ultra large endotoxin‐free plasmid extraction kits (Kangwei Century Biotechnology Co. LTD, CW2104); DMEM (Procell, PM150210); fetal bovine serum (ExCell Bio, FSP500); apoptosis detection kits (KGA108, Nanjing, China); 1% P/S (Procell, PB180120); and 0.25% Trypsin (BioFROXX, 1004GR025).

### 2.3 Establishment of an In Vitro Model of Pressure‐Injured Cell Damage

The pressure system included a high‐pressure cell culture device, CO_2_ pressure‐reducing valve, super constant temperature tank and steel cylinder. The experiments were conducted using an ultra‐clean workbench (China Suzhou Group Antai Air Technology Co. LTD., SW‐CJ‐1FD) and a CO_2_ incubator (SANYO, MCO‐15AC).

A stable temperature was maintained during the experiments using the super constant temperature tank (China Shanghai Pingxuan Scientific Instrument Co. Ltd.). High‐pressure cell culturing conditions were achieved using the PH‐PC‐01 cell high‐pressure culture device (Wuxi Pu He). For cell culture, DMEM medium (Procell, PM150210) was supplemented with fetal bovine serum (ExCell Bio, FSP500).

Briefly, the super thermostatic tank was filled with the appropriate amount of water and heated to 37°C. The CO_2_ pressure‐reducing valve was adjusted, and the exhaust switch of the high‐pressure culture unit was opened to fill the package with mixed gas (5% CO_2_ + 74% N_2_ + 21% O_2_). The exhaust switch was closed, and the intake switch on the CO_2_ pressure‐reducing valve was gradually adjusted until reaching the desired pressure (0.1 MPa, 0.8 MPa, 1,5 MPa, 2.0 MPa). Once pressurisation was complete, the thermostatic water tank was closed before turning off the cylinder knob. The high‐pressure device's exhaust switch was opened to slowly release gas. Finally, the pressure release valve was opened, and the petri dishes were collected for subsequent analysis.

### 2.4 Immunofluorescent Labelling

The slides were immersed in PBS, fixed with paraformaldehyde and permeated at room temperature with 0.5% Triton X‐100 for 20 min. Then, they were soaked in PBS, air‐dried, and treated with normal goat serum. The primary antibody was added, and the slides were incubated at 4°C overnight. Excess liquid was removed by soaking the slides in PBS, followed by air drying. The fluorescent secondary antibody was added to the slides, which were incubated at 37°C for 1 h. DAPI (4′,6‐diamidino‐2‐phenylindole) solution was added to the slides for an additional 5 min in darkness. The slides were sealed using an anti‐fluorescence quenching agent and observed under a fluorescence microscope equipped with an autofluorescence quencher. Image J software was utilised to analyse the fluorescence intensity of the images captured under 400× magnification.

### 2.5 Western Blotting

Proteins were extracted and separated using an SDS‐PAGE system and then transferred onto 0.45‐μm polyvinylidene difluoride (PVDF) membranes. Subsequently, the PVDF membranes were incubated overnight at 4°C with primary antibodies. After washing with buffer, the PVDF membranes were further incubated with peroxidase‐conjugated secondary antibodies. The signal was detected using the high‐sensitivity ECL Western Blotting substrate. Grey values of the film were analysed using Band Scan.

### 2.6 Assessment of Autophagy Flux Using a Double‐Labelled Adenovirus Harbouring Green (GFP) and/or Red Fluorescent Protein (RFP)‐Microtubule‐Associated Protein 1A/1B‐Light Chain 3 (LC3)

Plasmid transfection was performed according to the experimental groups as follows: Two hours prior to transfection, serum‐free DMEM medium was replaced with starvation medium. For each sample, 1.5 μg of Si‐TLR3 interference or TLR3 overexpression plasmid was diluted in 50 μL of serum‐free opti‐MEM and gently mixed for 5 min at room temperature. Lipofectamine 2000 was diluted in 50 μL of Opti‐MEM and incubated for 5 min at room temperature. Then, the diluted Lipofectamine 2000 and plasmid DNA solutions (total volume:100 μL) were gently mixed for 20 min at room temperature. The mixture was added to each well in the culture plate and shaken gently. The cells were incubated in a CO_2_ incubator at 37°C for 6 h, and then, the mixture was removed and replaced with normal medium. After an additional 6 h of culture, the cells were cultured in standard medium for 24 h. Subsequently, each well was supplemented with 1.5 μL of LBAD‐mCherry‐EGFP‐LC3 (10^10^ pfu/mL) adenovirus for 12 h. The cells were rinsed with PBS, fixed with paraformaldehyde and further soaked in PBS. Excess PBS was removed before dripping Hoechst33258 dye solution onto the slide and incubating it at room temperature in darkness. Once the liquid was evaporated entirely, anti‐fluorescent quenching agent was used to seal the film prior to image collection.

### 2.7 Measurement of Cell Proliferation Rates Using CCK‐8 Assay Kits

Treated cells were incubated with 10 μL of CCK‐8 per well at 37°C for 2 h. The absorbance at OD‐450 of each well was determined using an enzyme‐labelled assay, after which the percentage of cell proliferation was calculated.

### 2.8 Toluidine Blue Staining

Cell slides were immersed in PBS, fixed with paraformaldehyde, stained with 1% toluidine blue dye (preheated to 60°C), rinsed with distilled water, differentiated and decolorised using 95% alcohol. Then, they were examined under a microscope, dehydrated with anhydrous alcohol, clarified with xylene, air‐dried, sealed and photographed.

### 2.9 Flow Cytometric Analysis

Cells were treated with 0.25% pancreatic enzyme, collected and centrifuged. The supernatant was discarded, and the pellet was resuspended in PBS. The Annexin V‐FITC/PI apoptosis detection kit, ROS levels and JC‐1 dying solution were utilised to detect neuronal apoptosis, ROS, depolarisation of the mitochondrial membrane potential and mitochondrial membrane opening by flow cytometry, according to the manufacturer's instructions.

### 2.10 Data Analysis

All experimental data are presented as mean ± SEM (standard error of the mean). IBM SPSS Statistics 26 software was used to analyse the data. Two‐way analysis of variance (ANOVA) was performed, followed by multiple comparison tests. Pearson correlation coefficient was used to analyse the relationship between TLR3 and neuron death. Statistical significance was considered at *p* ≤ 0.05.

## 3 Results

### 3.1 Sustained Pressure Induces Neuronal Apoptosis and Neuronal Damage in VSC4.1 Cells

Currently, the in vivo animal models of compression spinal cord injury (SCI) were induced by several methods, including clip, balloon, solid spacer, expanding polymer, weight drop and strap [16]. In the previous reports, the pressures ranging from 0.2 megapascal (MPa) to 1 MPa were used to induce the compression SCI. The pressure of 100 pounds per square inch (PSI) (about 0.7 MPa) resulted in the abolish of the motor‐evoked potentials (MEPs) in hindlimb muscles in a yucatan pig model of SCI [17]. In a Sprague–Dawley rat model of SCI, the pressures of 100 kilodynes (kdyn) (about 0.2 MPa, converted based on the diameter of the impacts used in the experiment), 200 kdyn (converted to 0.4 MPa) and 300 kdyn (converted to 0.6 MPa) induced mild, moderate and severe spinal injury, respectively [18]. The user manual of the IH spinal cord impactor (Precision Systems and Instruments Inc., USA) recommends that for the mouse SCI model, the applied force ranges from 0.3 to 0.75 MPa to induce different degrees of SCI. However, the extents of the injury in animal models of SCI depend not only on the pressure but also on the speed, area, depth and duration of the impact. Therefore, there are few reports on the correlation between pressure and the severity of spinal cord injury.

To focus on a specific mechanism underlying the spinal injury, several in vitro models of SCI that mimic the in vivo spinal injury have been established by using motor neurons or cell lines. The injury in the cultured cells was induced by mechanical force (cut by scalpel) [19, 20] or by the prototypical endotoxin, lipopolysaccharide (LPS) [21]. However, the in vitro model of SCI that mimics the injury caused by compression has not yet been established.

**FIGURE 1:**
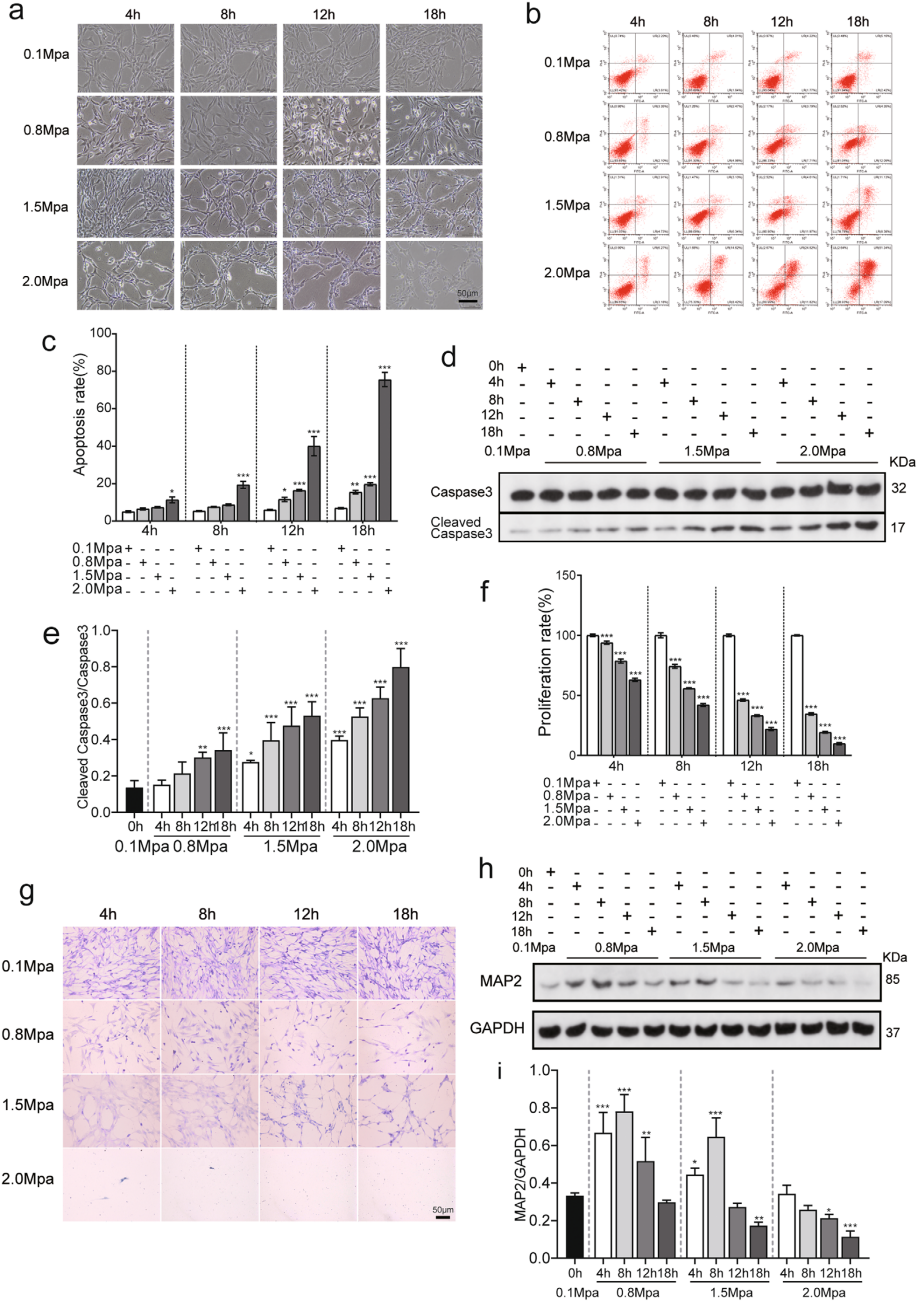
Sustained pressure induces neuronal apoptosis and neuronal damage in VSC4.1 cells. (a) Cellular morphological changes across various pressures and distinct pressure phases (400×). (b) Flow cytometric analysis of VSC4.1 cells at varied pressure conditions and compression protocols. (c) Assessment of cellular apoptotic rates in response to fluctuating pressure conditions across multiple pressure phases (*n* = 3). (d) Temporal evaluation via western blotting for Caspase‐3 and cleaved Caspase‐3 levels during sustained application of pressure. (e) Determination of the expression ratio between cleaved Caspase‐3 and Caspase‐3 in VSC4.1 cells subjected to varying magnitudes of applied pressure (*n* = 3). (f) Cellular proliferation in VSC4.1 cells across various applied pressures and duration intervals (*n* = 3). (g) Nissl staining of VSC4.1 cells after exposure to diverse pressure magnitudes and duration levels. (h) Western blot analysis of MAP2 expression in VSC4.1 cells subjected to sustained pressure at different intervals. (i) Immunofluorescence analysis of MAP2 expression levels in VSC4.1 cells under varying durations of sustained pressure (*n* = 3). Compared with 0.1 MPa, *p < 0.05, **p < 0.01, ***p < 005.

In the present study, a novel in vitro model of spinal cord compression injury was established by using VSC4.1 cells. Based on the previous studies, the pressures of 0.1 MPa (normal atmospheric pressure control), 0.8, 1.5 and 2.0 MPa were selected to treat the VSC4.1 cells. The cell morphology was observed under a microscope at 4, 8, 12 and 18 h after pressure treatment. In the 0.1 MPa group, the VSC4.1 cells showed a uniform distribution. However, the cells became less dense and exhibited swelling and disorderliness as the pressure levels and durations increased (Figure 1a).

To evaluate the effects of pressurisation on apoptosis levels, we performed Annexin V‐FITC/PI flow cytometry assays. The results demonstrate that under 0.8 and 1.5 MPa pressure, the VSC4.1 cells began to undergo apoptosis after 12 h pressure treatment, and that under 2.0 MPa pressure, the cells began to undergo apoptosis as early as 4 h after pressure treatment (Figure 1b,c, compared with 0.1 MPa, *p* < 0.05). At 2.0 MPa, the cells reached about 40% cell death at 12 h and 80% cell death at 18 h after pressure treatment.

To confirm that pressure induced cellular apoptosis of VSC4.1 cells, we evaluated the expression of cleaved caspase 3 by western blotting. The results showed that under 0.8 MPa pressure, cleaved caspase 3 levels were increased after 12 h pressure; and that under 1.5 and 2.0 MPa, cleaved caspase 3 levels were increased as early as 4 h, with additional increase over time after pressure (Figure 1d,e, compared with 0.1 MPa, *p* < 0.05).

To verify the neuronal damage caused by high pressure, we performed Cell Counting Kit‐8 (CCK‐8) assays. The proliferation rate was significantly reduced starting at 4 h after pressure in the 0.8, 1.5 and 2.0 MPa groups, and the degree of decline was proportional to the increasing pressure and time (Figure 1f,g).

For additional evidence of the effects of sustained pressure on VSC4.1 cells, we evaluated the expression of Microtubule‐Associated Protein 2 (MAP2), a marker of neural growth, axonal regeneration and synaptic plasticity. According to both western blotting and immunofluorescence staining assays, MAP2 expression was induced at 4 and 8 h after pressure treatment (compared with 0.1 MPa group, *p* < 0.05; Figure 1h,i); however, the levels returned to baseline at 12 and 18 h.

### 3.2 Sustained Pressure on VSC4.1 Cells Activates TLR3 Pathway Signalling

**FIGURE 2:**
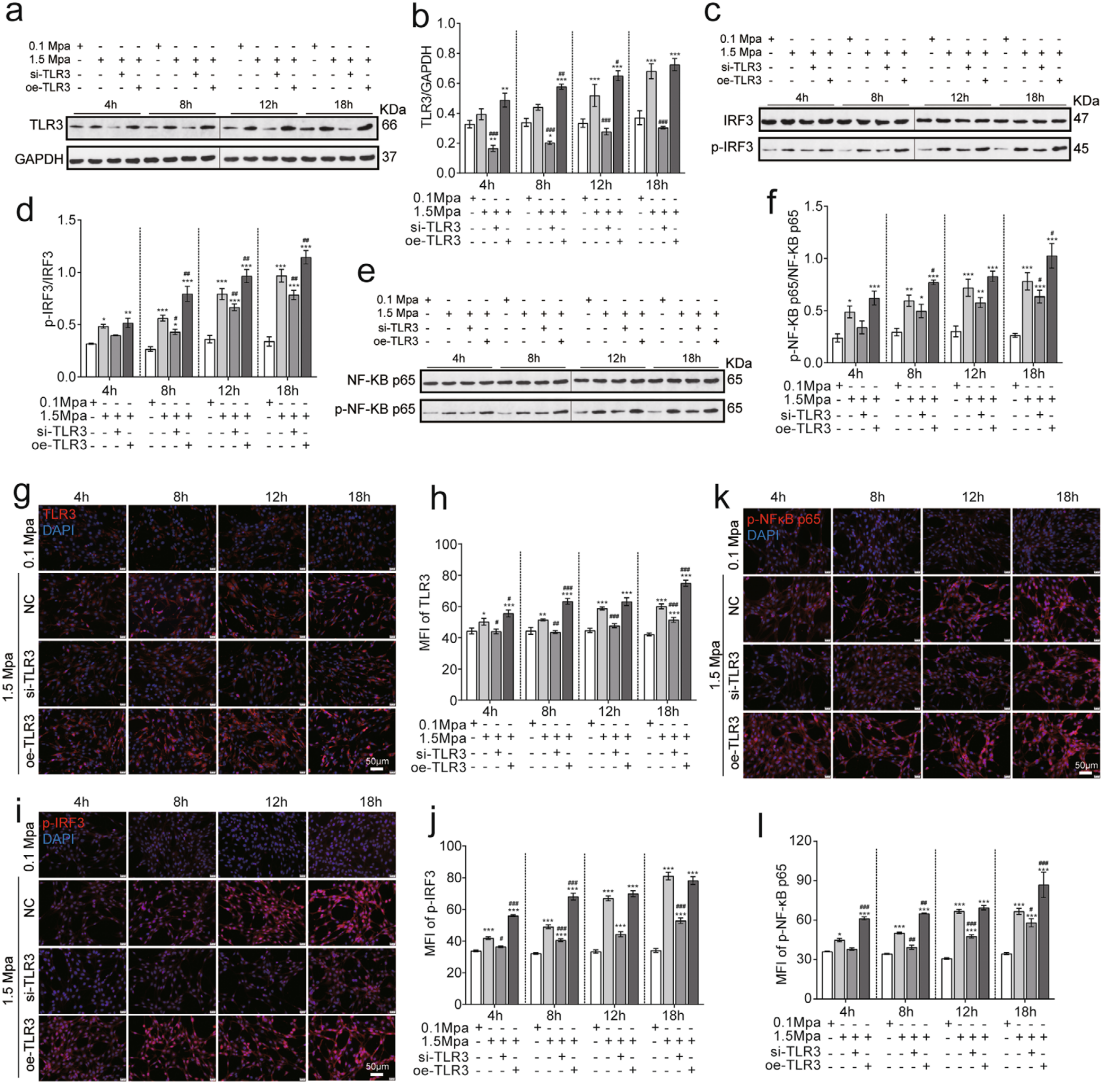
Sustained pressure on VSC4.1 cells activates TLR3 pathway signalling. (a) Western blot analysis of TLR3 in pressurised cells. (b) Quantification of the results from panel c (*n* = 3). (c) Western blot analysis IRF3 in pressurised cells. (d) Quantification of the results from panel e (*n* = 3). (e) Western blot analysis of NF‐κB p65 in cells subjected to pressure. (f) Quantification of the results from panel g (*n* = 3). (g) Immunofluorescence staining of TLR3 under pressure conditions. (h) Mean immunofluorescence intensity measurements for TLR3 staining in cells subjected to pressure (*n* = 3). (i) Immunofluorescence staining of phosphorylated IRF3 in pressure‐treated cells. (j) Mean immunofluorescence intensity measurements for phosphor‐IRF3 staining in pressure‐treated cells (*n* = 3). (k) Immunofluorescent staining of phosphorylated NF‐kB P65 in pressure‐treated cells. (l) Mean immunofluorescence intensity measurements for P‐NF‐KB P65 staining in pressure‐treated cells (*n* = 3). Compared with 0.1 MPa, *p < 0.05, **p < 0.01, ***p < 0.005; compared with 1.5 MPa, ^#^ *p* < 0.05, ^##^ *p* < 0.01, ^###^ *p* < 0.005. (scale bar = 20 μm).

To investigate the activation of the TLR3 pathway by mechanical pressure, we generated vectors for interfering with TLR3 (si‐TLR3) or overexpressing TLR3 (oe‐TLR3) (Figure S1), which were transfected into VSC4.1 cells prior to subjecting them to a pressure of 1.5 MPa for 4–18 h. The results demonstrate that pressure induced a time‐dependent increase in TLR3 levels, and the si‐TLR3 vector effectively reduced TLR3 levels below basal level, while the oe‐TLR3 vector significantly increased TLR3 levels in pressure‐induced cells at all time points (Figure 2a,b).

To verify that pressure activates the TLR3 pathway, we further measured the levels of the TLR3 signalling molecules IRF3 and NF‐κB P65 and their phosphorylated (activated) counterparts. The data demonstrate that 1.5 MPa promoted the phosphorylation of IRF3, which was reversed by si‐TLR3 and enhanced by oe‐TLR3 (Figure 2c,d). Similar results were observed for p‐NF‐κB P65/NF‐κB P65 (Figure 2e,f). These results were verified by immunofluorescence staining assays (Figure 2g–l), thus further confirming that sustained pressure activates the TLR3 signalling pathway.

To investigate the effect of TLR3 on neuron death, the correlation analysis between TLR expression and cleaved caspase‐3 as well as neuron death were performed. The results showed that there were positive correlations between TLR3 and cleaved caspase‐3 (*r* = 0.809, *p* < 0.01) and between TLR3 and apoptosis (*r* = 0.942, *p* < 0.01). The data demonstrated that the activation of TLR3 was closely related to the neuronal damage induced by continuous high pressure (Please see Figure S2e,g).

### 3.3 TLR3 Knockdown Mitigates Pressure‐Induced Neuronal Apoptosis

**FIGURE 3:**
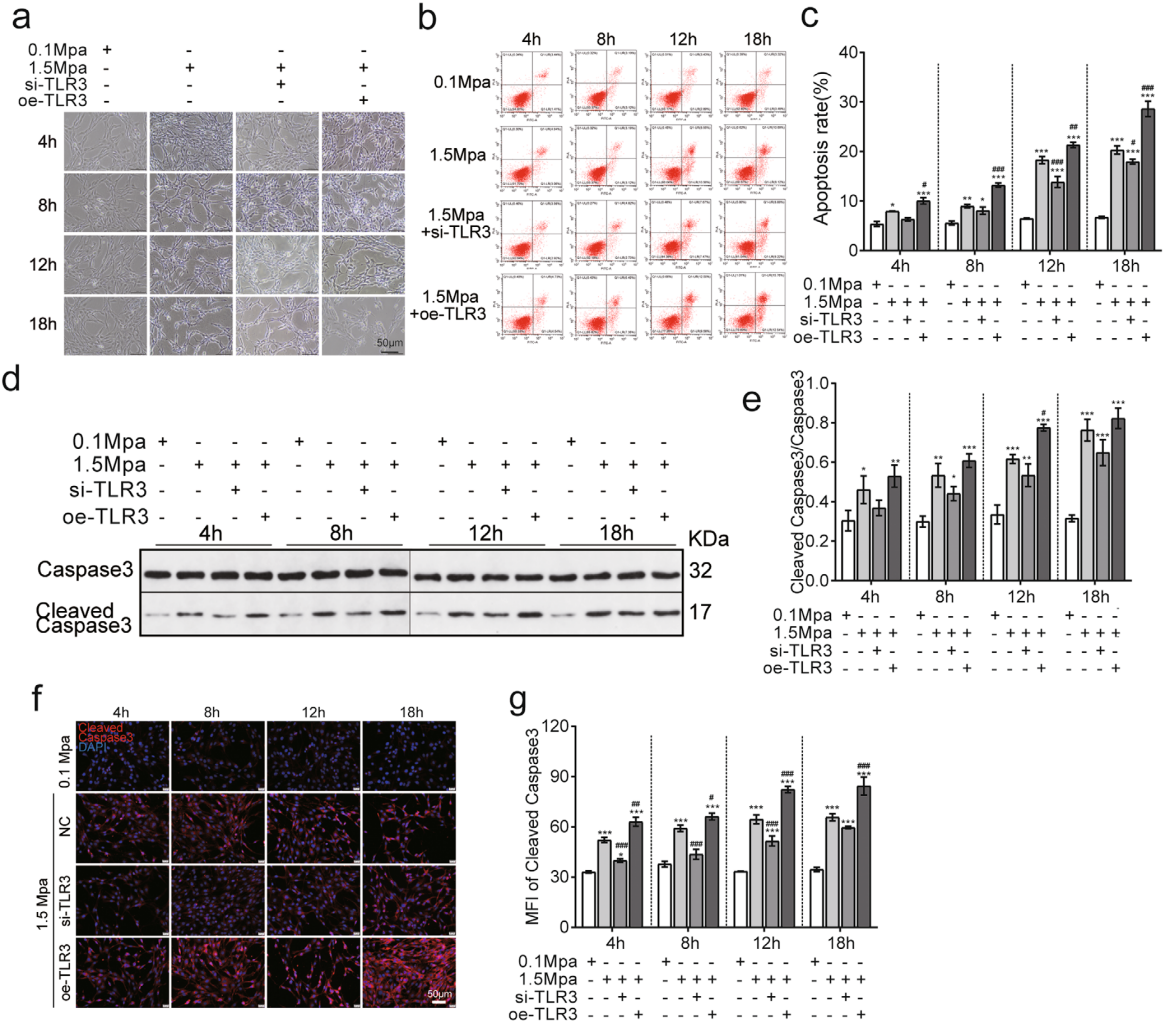
TLR3 knockdown mitigates pressure‐induced neuronal apoptosis. (a) Cell morphology observed using phase contrast microscopy after small molecule intervention targeting TLR3. (b) Flow cytometry analysis of the effects of sustained pressure and TLR3 knockdown or overexpression on VSC4.1 cells. (c) The apoptotic rate of VSC4.1 cells under sustained pressure after TLR3 knockdown or overexpression (*n* = 3). (d) Western blot analysis of the influence of TLR3 knockdown or overexpression on cleaved Caspase‐3 expression in VSC4.1 cells under sustained pressure. (e) The ratio of cleaved Caspase‐3 to Caspase‐3 expression, affected by TLR3 interference, was determined in VSC4.1 cells under sustained pressure (*n* = 3). (f) Evaluation of TLR3 knockdown or overexpression by immunofluorescence detection of cleaved Caspase‐3 in VSC4.1 cells subjected to sustained pressure. (g) Median fluorescence intensity measurement of cleaved Caspase‐3 (*n* = 3) in VSC4.1 cells with TLR3 knockdown or overexpression subjected to sustained pressure. Compared with 0.1 MPa, *p < 0.05, **p < 0.01, ***p < 0.005; compared with 1.5 MPa, ^#^ *p* < 0.05, ^##^ *p* < 0.01, ^###^ *p* < 0.005. The ‘+’represents knockout (si) or overexpression (oe). The ‘−’represents negative controls without knockout (si) or overexpression (oe). (scale bar = 20 μm).

To further evaluate the role of TLR3 in the response to pressure, we assessed the effects of siTLR3 on the cellular morphology of pressure induced VSC4.1 cells. The results demonstrate that siTLR3 alleviated the cellular dysfunction and oedema caused by pressure, while oe‐TLR3 exacerbated the effects of pressure in promoting cell damage (Figure 3a).

To further evaluate whether TLR3 expression regulates apoptosis, we determined the early apoptosis rates of VSC4.1 cells by flow cytometry. At each of the time points evaluated, the apoptosis rates were significantly higher in the si‐TLR3 group and significantly lower in oe‐TLR3 group than in the control 1.5 MPa‐treated group (*p* < 0.05, Figure 3b,c). Similar results were observed in the assessment of cleaved Caspase3 by western blotting (Figure 3d,e, *p* < 0.05) and immunofluorescence assay (Figure 3f, *p* < 0.05). Collectively, these results suggest that pressure‐induced VSC4.1 cell damage and apoptosis is TLR3‐dependent.

### 3.4 TLR3 Knockdown Protects VSC4.1 Cells From Neuronal Damage Caused by Sustained Pressure

**FIGURE 4:**
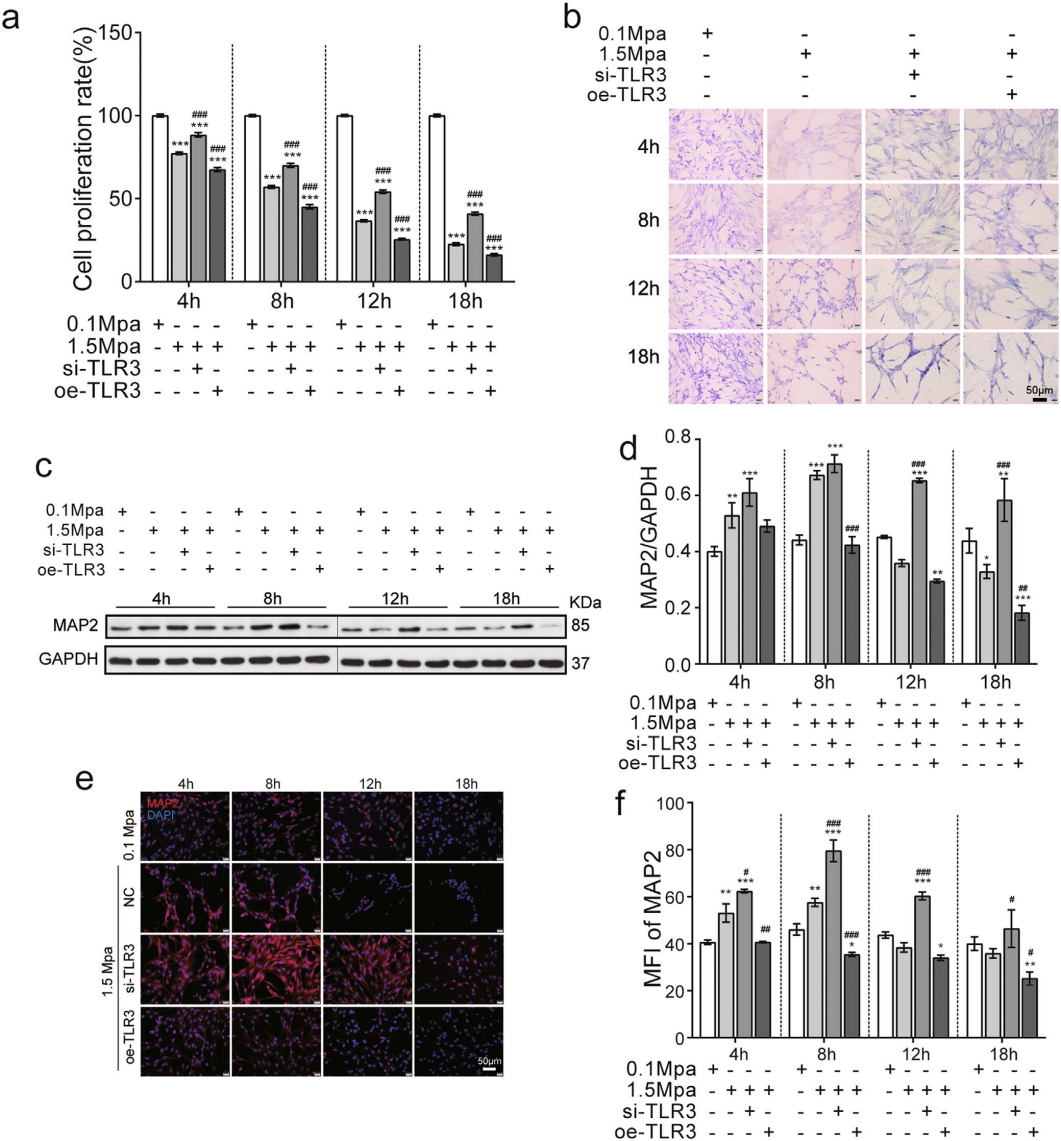
TLR3 knockdown protects VSC4.1 cells from neuronal damage caused by sustained pressure. (a) Impact of TLR3 knockdown on the proliferation rate of VSC4.1 cells under sustained pressure. (b) Visualisation of the effect of TLR3 knockdown by Nissl staining of VSC4.1 cells under sustained pressure. (c) Western blot analysis of MAP2 levels upon TLR3 knockdown in VSC4.1 cells subjected to sustained pressure. (d) Influence of TLR3 knockdown on MAP2 expression in VSC4.1 cells under sustained pressure. (e) TLR3 knockdown alters MAP2 immunofluorescence in VSC4.1 cells exposed to sustained pressure. (f) TLR3 knockdown affects the median fluorescence intensity of MAP2 in VSC4.1 cells under sustained pressure (*n* = 3). Compared with 0.1 MPa, *p < 0.05, **p < 0.01, ***p < 0.005; compared with 1.5 MPa, ^#^ *p* < 0.05, ^##^ *p* < 0.01, ^###^ *p* < 0.005. The ‘+’represents knockout (si) or overexpression (oe). The ‘−’represents negative controls without knockout (si) or overexpression (oe). (scale bar = 20 μm).

To further evaluate the effect of TLR3 modulation on pressure‐induced neuronal cell damage, we measured proliferation rates in si‐TLR3 and oe‐TLR3 VSC4.1 cells by CCK8 assay. The results indicate that cell proliferation rates were significantly reduced at 4 h after treatment with 1.5 MPa, as compared to the rates in the 0.1 MPa control group; moreover, si‐TLR3 significantly reversed the effects of pressure treatment on VSC4.1 cell proliferation, while oe‐TLR3 significantly exacerbated the effects of pressure treatment (*p* < 0.05, Figure 4a), and Nissl staining revealed shorter or thinner with shallower stained cells for all high pressure‐treated cells (Figure 4b).

To further examine the effects of TLR3 expression on the expression of MAP2, we performed western blotting assays. While MAP2 expression was increased at 4 and 8 h after pressure treatment (1.5 MPa vs. 0.1 MPa, *p* < 0.05), this effect was enhanced in the si‐TLR3 group and inhibited in the oeTLR3 group (*p* < 0.05, Figure 4c,d). The results were verified by immunofluorescence assay (Figure 4e,f). As MAP2 serves as a marker of neural growth [22], these findings indicate a potential mechanism by which downregulation of TLR3 protects neural cells from sustained pressure.

### 3.5 TLR3 Knockdown Enhances the Induction of Protective Autophagy in VSC4.1 Cells Under Sustained Pressure

**FIGURE 5:**
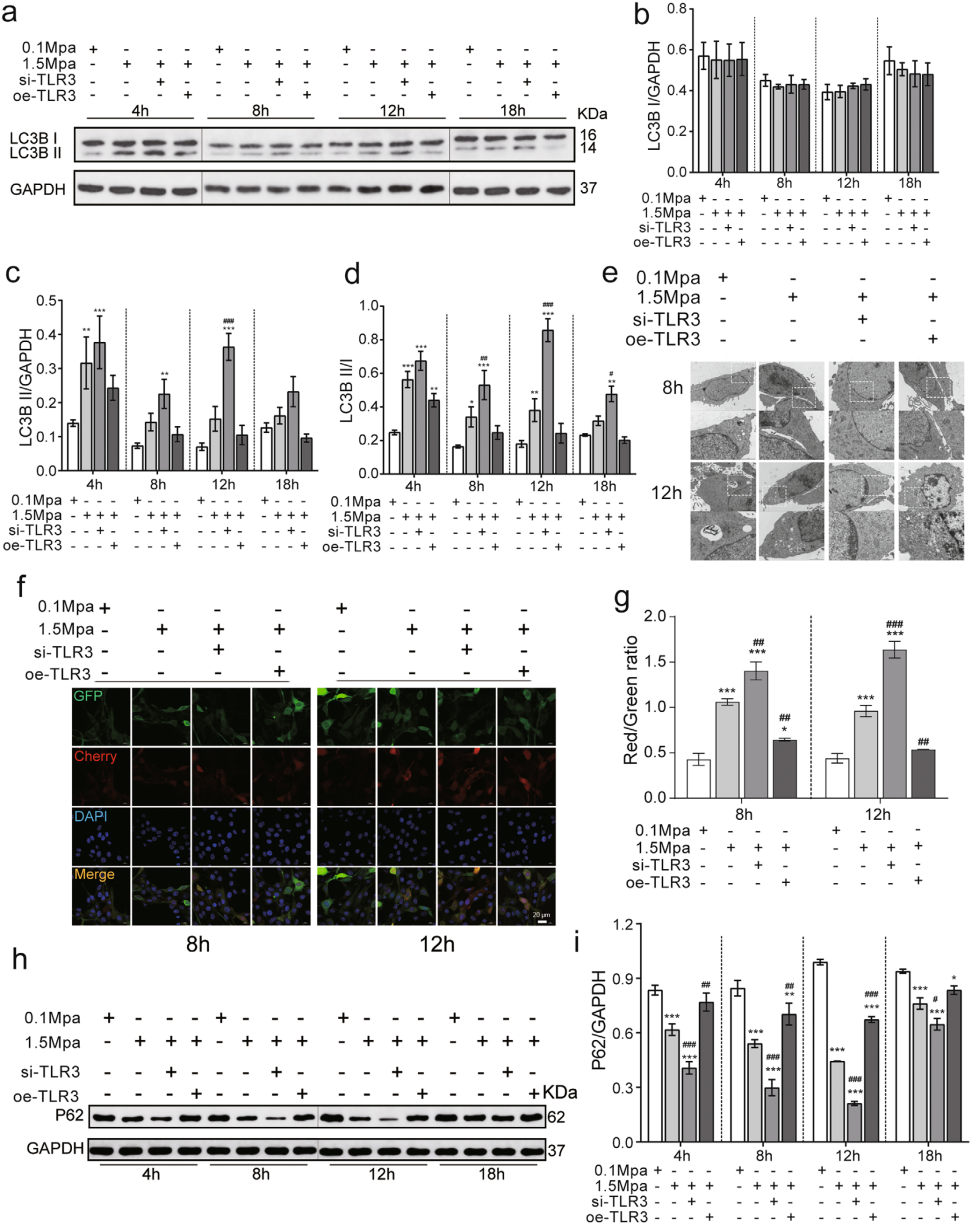
TLR3 knockdown enhances the induction of protective autophagy in VSC4.1 cells under sustained pressure. (a) Western blot analysis of LC3B expression in the VSC4.1 in vitro spinal cord stress injury model. (b) Quantification of LC3B‐I levels in VSC4.1 cells subjected sustained pressure (*n* = 3). (c) Quantification of LC3B‐II levels in VSC4.1 cells subjected to sustained pressure (*n* = 3). (d) Calculation of the ratio between LC3B‐II and LC3B‐I expression levels in VSC4.1 cells under sustained pressure (*n* = 3). (e) Autophagy structures and mitochondria were detected by transmission electron microscopy. (f) Visualisation of autophagy flux using Ad‐mCherry‐GFP‐LC3 in VSC4.1 cells. Bright yellow spots upon infection with lentiviruses carrying RFP and GFP markers indicate the occurrence of autophagy. Upon fusion with lysosomes, the decreased pH leads to quenching of modified GFP protein fluorescence, resulting in red light only. (g) The ratio of the average fluorescence intensity of red/green light was calculated to measure the number of autophagic lysosomes (*n* = 3). (h) Western blot analysis of P62 expression in the VSC4.1 in vitro spinal cord stress injury model. (i) Quantification of P62 levels in VSC4.1 cells subjected sustained pressure (*n* = 3). Compared with 0.1 MPa, *p < 0.05, **p < 0.01, ***p < 0.005; compared with 1.5 MPa, ^#^ *p* < 0.05, ^##^p < 0.01, ^###^ *p* < 0.005. The ‘+’represents knockout (si) or overexpression (oe). The ‘−’represents negative controls without knockout (si) or overexpression (oe). (scale bar = 20 μm).

Microtubule‐associated protein 1A/1B‐light chain 3 (LC3B) serves as an autophagic marker. LC3B‐I is localised to the cytoplasm, while LC3B‐II is a converted and membrane‐bound form of LC3B‐I that localises on vacuoles and initiates the formation and lengthening of the autophagosomes [23]. Therefore, we analysed the expression of LCB‐I and LC3B‐II to evaluate the effects of TLR3 modulation on autophagy. As shown in Figure 5a–d, the LC3B‐II levels and the ratio of LC3B‐II/LC3B‐I in VSC4.1 cells were significantly increased starting at 4 h after pressure treatment (1.5 MPa vs. 0.1 MPa, *p* < 0.05). Additionally, the si‐TLR3 group showed a further significant increase in LC3B‐II and LC3B‐II/LC3B‐I (siTLR3 vs. 1.5 MPa, *p* < 0.05), while the oe‐TLR3 group showed no significant difference (oeTLR3 vs. 1.5 MPa, *p* > 0.05).

For additional verification, we used transmission electron microscopy to observe the ultrastructure of VSC4.1 cells. As shown in Figure 5e, the 0.1 MPa group exhibited a nucleus with a complete, oval or round membrane, evenly distributed chromatin and abundant organelles. Most mitochondria had elongated or oval shapes with intact membranes. However, in the 1.5 MPa group, mitochondria appeared significantly swollen with a densely arranged network structure, occasionally accompanied by sporadic autophagic structures. In contrast, the 1.5 MPa si‐TLR3 group exhibited rounded mitochondria with a more evenly distributed autophagy structure, fewer autophagosomes, some chromatin condensation, reduced boundary definition and decreased apoptotic bodies, while the 1.5 MPa oe‐TLR3 group showed swollen mitochondria aligned along the long axis of the cell membrane with highly irregular and fragmented mitochondrial ridges, an increased number of autophagosomes and occasional instances of structured autophagy.

Finally, we used Ad‐mCherry‐GFP‐LC3 to visualise the autophagosomes in pressure‐treated VSC4.1 cells (Figure 5f). The results indicate that si‐TLR3 significantly increased both the number of autophagolysosomes and the number of autophagy events (Figure 5g) at 8 and 12 h after pressure treatment (*p* < 0.05). Collectively, these results indicate that sustained pressure of VSC4.1 cells induces autophagy, and TLR3 knockdown enhances the induction of protective autophagy in VSC4.1 cells under sustained pressure.

### 3.6 TLR3 Knockdown Increases Mitochondria‐Associated Autophagy

**FIGURE 6:**
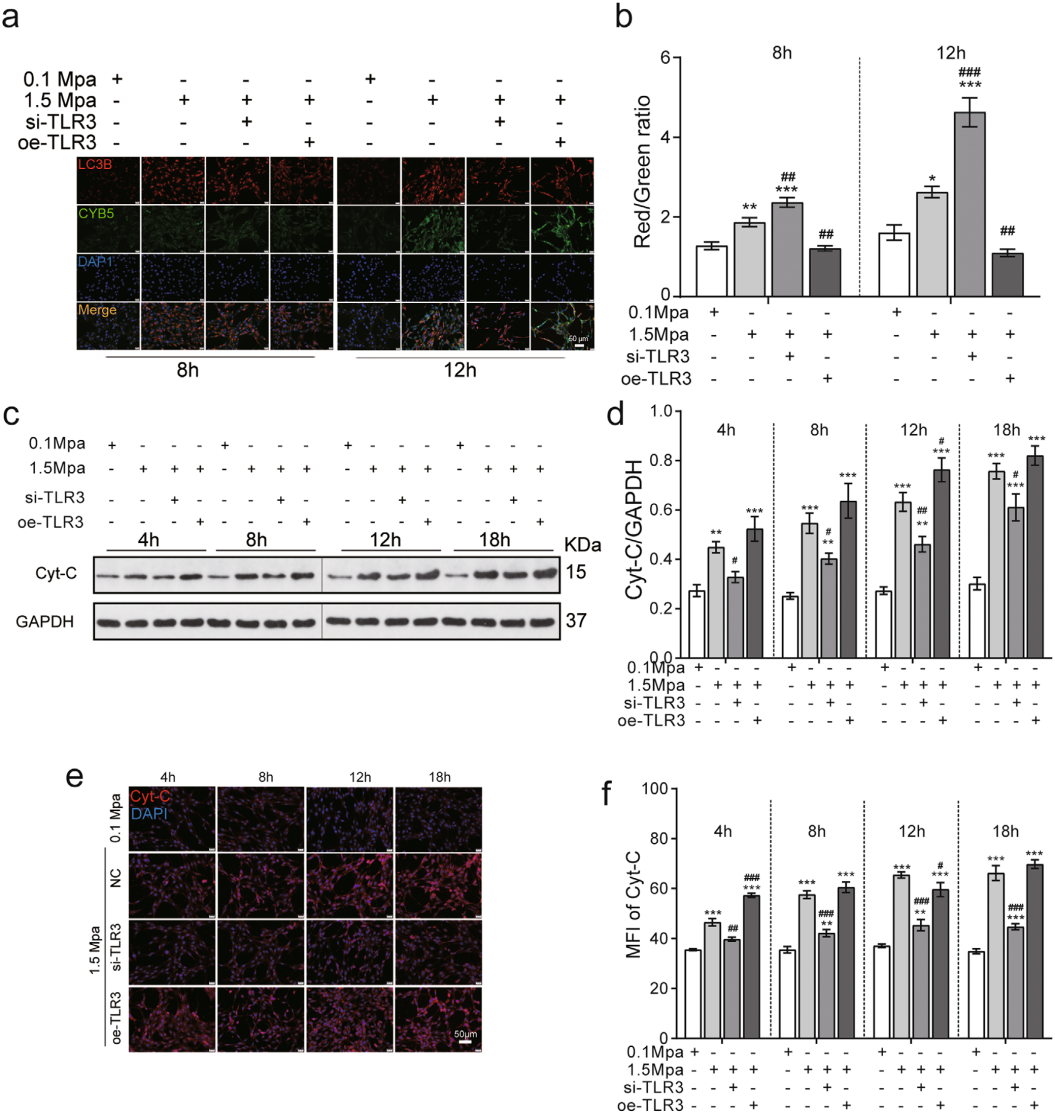
TLR3 knockdown increases mitochondria‐associated autophagy. (a) The relationship between autophagy and mitochondria was evaluated using LC3‐COXIV colocalised immunofluorescence staining (*n* = 3). DAPI was used as a background stain. (b) The ratio of red/green mean fluorescence intensity was measured in LC3‐COXIV immunofluorescence colocalisation (*n* = 3). (c) Western blotting of the expression level of Cyt‐c under pressure (*n* = 3). (d) The expression level of Cyt‐c was evaluated in pressure‐treated cells (*n* = 3). (e) Cyt‐c immunofluorescence was employed to assess mitochondrial damage in pressure‐treated cells. (f) The median fluorescence intensity of Cyt‐c was measured in pressure‐treated cells (*n* = 3). Compared with 0.1 MPa, *p < 0.05, **p < 0.01, ***p < 0.005; compared with 1.5 MPa, ^#^ *p* < 0.05, ^##^ *p* < 0.01, ^###^ *p* < 0.005. The ‘+’represents knockout (si) or overexpression (oe). The ‘−’represents negative controls without knockout (si) or overexpression (oe). (scale bar = 20 μm).

Mitophagy, a specialised process involving the degradation of damaged mitochondria within autophagosomes, is characterised by the colocalisation of LC3B and CYB5 on the outer membrane of mitochondria. To investigate whether sustained pressure on VSC4.1 cells promotes mitochondria‐associated autophagy, we conducted immunofluorescence assays. The results demonstrate that pressure treatment (1.5 MPa for 8 and 12 h) enhances LC3B‐CYB5 colocalisation, which is further enhanced by si‐TLR3 and conversely inhibited by oe‐TLR3 (Figure 6a,b, *p* < 0.05). Cytochrome c (Cyt‐c), an essential component involved in mitochondrial electron transport and intrinsic type II apoptosis protein, was evaluated through Western blotting and immunofluorescence assays to further assess the impact of sustained pressure on the mitochondrial stress response. The findings reveal that Cyt‐c expression significantly increases after exposure to 1.5 MPa pressure for 4 to 18 h; however, si‐TLR3 reverses this elevation in Cyt‐c expression (Figure 6c–f). These results indicate that TLR3 knockdown increases mitochondria‐associated autophagy and the mitochondrial stress response.

Selective autophagy degrades the aggregated proteins and also removes the unnecessary or impaired mitochondria [24, 25, 26]. Sequestosome‐1 (p62), a classical receptor of autophagy, is a multifunctional protein located throughout the cell. It is involved in many signal transduction pathways and widely used as a predictor of autophagic flux. Previous study reported that activating autophagy reduced the expression of p62, on the contrary, pharmacological and genetic inhibition of autophagy increased the level of p62 in various cell lines [27]. As shown in the Figure 5h,i, compared with controls, the levels of p62 in pressure treatments groups were significantly reduced (*p* < 0.05). Interestingly, the siTLR3 further decreased, whereas oeTLR3 increased the levels of p62 in all the time points after pressure treatment (*p* < 0.05).

### 3.7 TLR3 Knockdown Ameliorates Mitochondrial Dysfunction in VSC4.1 Cells Exposed to Sustained Pressure

**FIGURE 7:**
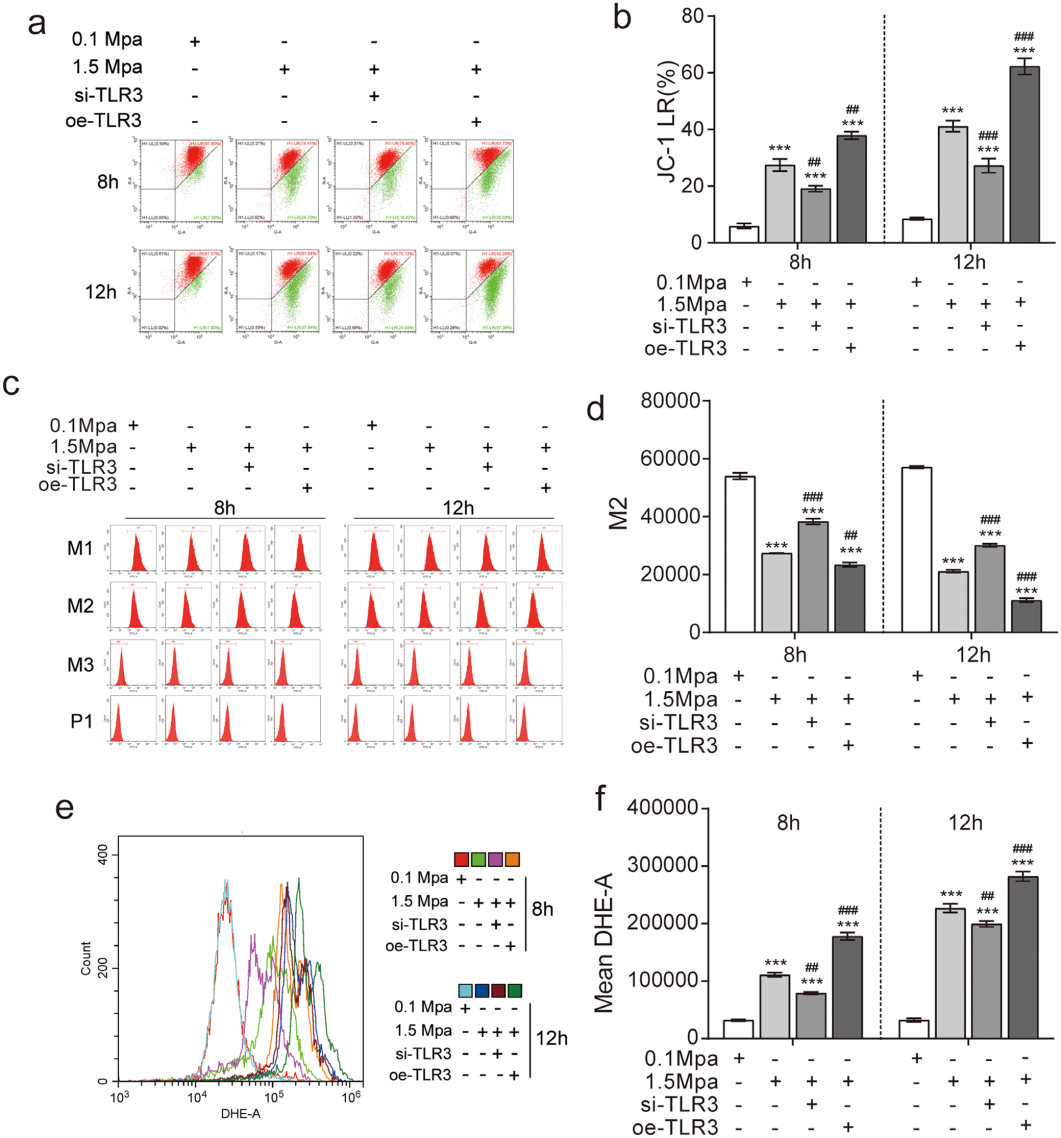
TLR3 knockdown ameliorates mitochondrial dysfunction in VSC4.1 cells exposed to sustained pressure. (a) JC‐1 staining results showing the cellular response to pressure (*n* = 3). JC‐1 accumulates within the matrix at high mitochondrial membrane potential, forming J‐aggregates that emit red fluorescence. Conversely, JC‐1 exists as monomers emitting green fluorescence at low mitochondrial membrane potential. (b) Mean intensity analysis of green fluorescence emitted by JC‐1 monomers localised within mitochondria under pressure conditions (*n* = 3). (c) Mitochondrial MPTP opening rate of pressurised cells (*n* = 3). M1: High fluorescence signal of the cytoplasm and mitochondria; M2: Mitochondrial fluorescence signal only. M1‐M2 indicates the high fluorescence signal in the cytoplasm. (d) High fluorescence signal M2 (*n* = 3) in mitochondria of pressurised cells. (e) DHE‐A assay of mitochondrial reactive oxygen species (ROS) during stress injury (*n* = 3). (f) Quantification of the results in panel e (*n* = 3). Compared with 0.1 MPa, *p < 0.05, **p < 0.01, ***p < 0.005; compared with 1.5 MPa, ^#^ *p* < 0.05, ^##^ *p* < 0.01, ^###^ *p* < 0.005. The ‘+’represents knockout (si) or overexpression (oe). The ‘−’represents negative controls without knockout (si) or overexpression (oe).

Next, to compare mitochondrial transmembrane potential depolarisation, we performed JC‐1 staining assays. The JC‐1 monomer (LR) was significantly increased, and the JC‐1 aggregate (UR) was significantly decreased at 8 and 12 h after pressure treatment (*p < 0.05, 1.5 MPa vs. 0.1 MPa group); furthermore, these changes in response to high‐pressure treatment were less dramatic in the si‐TLR3 group but were exacerbated in the oe‐TLR3 group (^#^ *p* < 0.05), compared to the untreated 1.5 MPa group (Figure 7a,b).

For additional confirmation, we evaluated the M2 (mitochondrial) hyper‐fluorescence signals to assess the opening rate of the mitochondrial permeability transition pore. Compared to the 0.1 MPa control group, the 1.5 MPa group displayed a significant decrease in both the M2 signals at 8 and 12 h. Moreover, this decrease was reversed by si‐TLR3 treatment, while oe‐TLR3 treatment had the opposite effect (Figure 7c,d).

Finally, we performed DHEA assays to measure the levels of reactive oxygen species (ROS). In comparison with the 0.1 MPa group, the 1.5 MPa group displayed significant increases in ROS at 8 and 12 h, and this increase was partially reversed in the si‐TLR3 and increased in the oe‐TLR3 group (Figure 7e,f). Collectively, these findings support pressure‐induced mitochondrial dysfunction can be ameliorated by using TLR3 siRNA.

## 4 Discussion

SCI is a devastating neurological condition that has tripled in global prevalence over the past three decades [28]. Acute SCI commonly occurs when the spinal cord is suddenly compressed due to trauma‐induced fractures and vertebral dislocation. This primary injury may result in the destruction of neural parenchyma, disruption of axonal network and a subsequent cascade of events associated with secondary injury [29]. To investigate the mechanism, pathology and treatment of spinal cord injury, a variety of in vivo animal models and in vitro spinal cord injury models have been established. However, to our knowledge, this is the first study to establish in vitro model for injury caused by compression.

External mechanical stress can induce pathogenic diseases, which depend on the severity, location and duration of exposure [30, 31, 32]. In the present study, a thermostatic incubator combined with pressure gas tank and VSC4.1 cells were utilised to simulate SCI‐associated pressure‐induced cell damage across a range of severity. Our data confirm that as the pressure levels and durations increased, the cell morphology changed from uniform distribution to less dense, with increased swelling and disorderliness. Consistent with pathogenic disease progression, apoptosis in VSC4.1 cells was induced by pressure treatment in a time and dose‐dependent manner, which was confirmed both by V‐FITC/PI flow cytometry assay and assessment of cleaved Caspase 3 levels. Therefore, our in vitro model recapitulates the effects of mechanical stress in response to varying levels of severity.

Previous studies indicated that the primary damages of traumatic brain injury (TBI) involve a complex interplay of forces, including impact loading, blast overpressure and impulsive loading [33]. Mass effect is a major mechanism of primary brain injury after intracerebral haemorrhage (ICH). Mass effect directly formed by hematoma can mechanically compress and stretch the neurons and glial cells of surrounding brain tissues, leading to the physical destruction of neural network structure [34]. The primary damages result in diverse injury patterns, such as oxidation stress, hematoma degradation and inflammation activation, which are so called the secondary brain injury following TBI and ICH. In addition, in the growth of a brain tumour, the mass effect is also a major cause of neurologic injury and affects tumour progression [35]. However, the detailed pathologic cascades have not been fully elucidated. Currently, the in vitro models mimicking the pressure‐induced damage for TBI, ICH and tumour mass effect have not been reported. Based on the replication of apoptotic, autophagic and mitochondrial processes within our model, it may be useful for in vitro studies of diverse acute and chronic compression injuries, including but not limited to spinal cord injury, traumatic brain injury, intracranial hematoma and tumours.

As shown in Figure 1, the data demonstrated that under the pressure of 2.0 MPa, significant neuronal apoptosis occurred 4 h after treatment, and the apoptosis rate reached over 75% after 18 h. Under the pressures of 0.8 and 1.5 MPa, neuronal apoptosis began to occur at 12 h after treatment. On the other hand, the cleaved caspase‐3 significantly increased at 4 h after treatment under 2.0 MPa, at 8 h under 1.5 MPa and at 12 h under 0.8 MPa. Based on the neuronal apoptosis rates and levels of cleaved capsase‐3, and considering the interference intensity of the experiment, a pressure of 1.5 MPa was selected for subsequent experiments, because the interference intensity is relatively larger at a pressure of 2.0 MPa and smaller at a pressure of 0.8 MPa.

In the present study, the role of TLR3 in regulating the biological effects of sustained pressure treatment was investigated. The effects of si‐TLR3 and oe‐TLR3 on neurons under 0.1 MPa were detected. The data showed that compared with the control groups, the level of TLR3 significantly increased in oe‐TLR3 neurons and decreased in the si‐TLR3 neurons (Figure S1l,m).

TLR3 belongs to a family of transmembrane proteins that play an instructive role in innate and adaptive immune responses [36], and excessive activation of TLR signals may lead to hyperactive immunity as an intrinsic cause associated with immune factors [37, 38]. Furthermore, the TLR3‐mediated signalling pathway has been reported to contribute to neuroinflammation and related nerve system diseases [39].

TLR3 is expressed both on the cell surface and intracellularly, such as endosomal compartment of cells. Unlike other TLR family members, TLR3 is the only RNA sensor that is utterly dependent on the adaptor protein TRIF. TLR3 binds to its ligand, double‐stranded RNA (dsRNA), and then activates the TRIF pathway, mediating the transcriptional induction of type I interferons (IFNs), proinflammatory cytokines and chemokines. The dsRNA is produced not only by viral replication but also by the necrotic, apoptotic and otherwise stressed cells [40], including the uptake of apoptotic bodies, clathrin dependent endocytosis and autophagic uptake of dsRNA from the cytosol [41].

DsRNA accumulation potentially activates the innate immune system and promotes neuroinflammation. Research on amyotrophic lateral sclerosis (ALS) demonstrated that the animals with heavy dsRNA expression showed markedly increased levels of astrogliosis and microgliosis contributing to the degeneration of spinal cord neurons [42]. Synthetic TLR3 ligand poly I:C is frequently used to investigate the innate immune responses in models of psychiatric and neurodegenerative disorders including schizophrenia, autism, Parkinson's disease and Alzheimer's disease. The report indicated that poly I:C produced sickness behaviour and robust IL‐6, IFN‐I and TNF‐α responses [43]. Human neurons also express TLR‐3 and can mount a strong inflammatory response characterised by the expression of inflammatory cytokines, chemokines and antiviral molecules after treatment with dsRNA, which indicates that human neurons, in absence of glia, have the intrinsic machinery to trigger robust inflammatory, chemo attractive and antiviral responses [44]. Our preliminary experiments revealed that the content of dsRNA increased in the neurons subjected to the pressure treatment compared to the normal control (Please see Figure S2h).

The data of the present study showed that TLR3 and its downstream pathways were upregulated and activated. A possible explanation for this phenomenon is that the high pressure induced VSC4.1 cell damage, and the injured neurons released damage‐associated molecular pattern molecules (DAMPs) including dsRNA. The DAMPs bind to TLR3 as ligands and activated TLR3. The activation of TLR3 induced the secretion of interferons and the phosphorylation and translocation of NF‐κB through TRIF‐dependent pathway. Our data also demonstrated that there were positive correlations between TLR3 and cleaved caspase‐3 (*r* = 0.809, *p* < 0.01) and between TLR3 and apoptosis (*r* = 0.942, *p* < 0.01), which indicated that the activation of TLR3 was closely related to the neuronal damage induced by continuous high pressure (Please see Figure S2e,g). Collectively, these results suggest that pressure‐induced VSC4.1 cell damage and apoptosis is TLR3‐dependent. However, the present study primarily focused on the receptor (TLR3) and its pathway but not on its ligands. Further investigation on the intrinsic ligands of TLR3 in the pressure‐induced damaged neurons will be of great help in understanding the mechanism of TLR3 signalling pathway involved in SCI. In addition, research on the effect of specific antagonists for TLR3, such as CU‐CPT4a and TLR3/dsRNA Complex Inhibitor, on pressure‐induced neuron injury will provide potential targets for the treatment of SCI.

In our in vitro model of compression, the expression of TLR3 and the phosphorylation of its downstream targets (pIRF3 and p‐NF‐κB) were upregulated by pressure treatment. The siTLR3 reduced the levels of pIRF3 and p‐NF‐κB, as well as several additional important molecular targets, including the mitochondrial protein Cyt‐c and the autophagy protein LC3BII/I. These findings support a role for TLR3 in mediating pressure‐induced mitochondrial dysfunction and autophagy. The presented by p65 also indicated that selective autophagy was activated in pressure‐induced damaged neurons. The siTLR3 enhanced autophagy whereas oeTLR3 inhibited autophagy. Although our data revealed a link between the autophagy and pressure‐induced damage, further investigation is required to make a final affirmative determination.

Our data demonstrated that mechanical pressure reduced the cell viability, as evaluated by CCK‐8 assay. The siTLR3 attenuated the decrease in cell viability induced by pressure treatment, whereas oe‐TLR3 aggravated the decrease in cell proliferation (Figure 4a,b). Interestingly, MAP2, a marker for neuronal differentiation, increased at earlier time points (4 and 8 h) and decreased at later timepoints (12 and 18 h) after high‐pressuring treatments. Microtubule‐associated protein‐2 (MAP‐2) is a family of heat‐stable, phosphoproteins expressed predominantly in the cell body and dendrites of neurons. MAP‐2 plays an important role in microtubule dynamics to maintain neural architecture. In addition, MAP‐2 is exquisitely sensitive to many inputs and has been confirmed to have dynamic functions in the growth, differentiation and plasticity of neurons [45, 46]. In our experiment, the upregulation of MAP‐2 in the early time points (4 and 8 h) reflected the compensatory mechanism of neurons. In response to the pressure treatment, the expression of MAP‐2 increased to protect neurons from damage. The downregulation of MAP‐2 in the late stage (12 and 18 h) indicated damage to the neural architecture. Our data showed that si‐TLR3 significantly increased MAP‐2 levels and maintained it at a high level in the late stage, whereas oe‐TLR3 significantly decreased MAP‐2 levels at the time point of 8, 12 and 18 h (Figure 4c,d). The results indicated that inhibition of TLR3 protected neurons from pressure‐induced damage by increasing MAP‐2 expression, and that overexpression of TLR3 had the opposite effects.

We also demonstrated a role for si‐TLR3 in decreasing the mitochondrial permeability transition pore (MPTP) potential. A decrease in mitochondrial potential leads to the opening of membrane pores and increased permeability, releasing apoptosis initiator factors into the cytoplasm [47, 48, 49]. Mitochondrial Cyt‐c release plays a crucial role in activating cell death [50, 51, 52]. Water‐soluble protein Cyt‐c acts as a marker for damage [53, 54]. Similarly, under 1.5 MPa pressure, VSC4.1 cell mitochondria showed enlarged inner compartments with concentrated contents and swollen outer compartments that appeared aggregated. The increased depolarisation and peroxidation of the mitochondrial membrane led to a higher opening rate of the membrane pore protein, though si‐TLR3 significantly alleviated these effects, thus verifying its function in regulating MPTP function.

The timely removal of damaged mitochondria through autophagy is considered a cellular self‐protective mechanism, and dysfunction of mitochondrial autophagy can trigger various diseases [55, 56, 57]. LC3 is typically localised to the cytoplasm during autophagy induction and remains within the autophagic structure until late‐stage autolysosomes [58, 59]. Cytochrome b5 localises to the outer mitochondrial membrane and colocalises with the autophagy marker LC3, suggesting that mitochondrial autophagy originates from this double‐layered membrane structure [60, 61, 62]. In our VSC4.1 pressure model, the expression level of LC3BII/I increased at 1.5 MPa VSC4.1, and Cytochrome b5 colocalised with LC3B. However, in si‐TLR3 cells, there was a significant decrease in cytochrome C expression accompanied by an increase in LC3BII/I protein levels that promoted the colocalisation of Cytochrome b5 with LC3B. As a result, RNAi targeting TLR3 mitigated mitochondrial damage and enhanced autophagy to facilitate cellular repair under pressure.

In summary, we established a novel in vitro model of pressure‐induced neuron damage. TLR3, IRF3 and NF‐κB were activated in VSC4.1 cells subjected to high‐pressure treatment. TLR3 blockage via siRNA attenuated VSC4.1 cell damage, while TLR3 overexpression aggravated the cellular damage. The mechanisms underlying the effect of TLR3 on VSC4.1 cell damage induced by high‐pressure may be related to its role in the regulation of mitochondrial function and autophagy.

## Author Contributions

**Li Lin:** conceptualization (lead), data curation (equal), formal analysis (equal), investigation (equal), methodology (equal), project administration (equal), resources (equal), software (equal), supervision (equal), validation (equal), visualization (equal), writing – original draft (equal), writing – review and editing (equal). **Zhongzhong Lv:** data curation (equal), formal analysis (equal), investigation (equal), resources (equal), software (equal), supervision (equal), validation (equal), visualization (equal), writing – original draft (equal). **Chao Zhou:** data curation (equal), formal analysis (equal), investigation (equal), methodology (equal), project administration (equal), resources (equal), software (equal), supervision (equal), validation (equal), visualization (equal), writing – original draft (equal), writing – review and editing (equal). **Taiyang Zhu:** data curation (equal), formal analysis (equal), investigation (equal), methodology (equal), project administration (equal), supervision (equal), validation (equal), visualization (equal), writing – original draft (equal). **Yuting Hu:** data curation (equal), formal analysis (equal), investigation (equal), methodology (equal), project administration (equal), resources (equal), software (equal), supervision (equal), validation (equal), visualization (equal). **Xiaoyu Sun:** data curation (equal), formal analysis (equal), investigation (equal), methodology (equal), project administration (equal), resources (equal). **Hui Zhou:** resources (equal), software (equal), supervision (equal), validation (equal), visualization (equal). **Miao Wang:** data curation (equal), project administration (equal), software (equal), validation (equal), visualization (equal). **Yongtao Lin:** data curation (equal), software (equal), validation (equal), visualization (equal). **Guoqing Gu:** resources (equal), software (equal), supervision (equal), validation (equal), visualization (equal). **Shang Wang:** data curation (equal), software (equal), validation (equal), visualization (equal). **Yan Zhou:** resources (equal), validation (equal), visualization (equal). **Jingjing Han:** resources (equal), validation (equal), visualization (equal). **Guoliang Jin:** software (equal), validation (equal), visualization (equal). **Fang Hua:** conceptualization (equal), funding acquisition (lead), investigation (equal), project administration (equal), resources (equal), writing – review and editing (equal).

## Conflicts of Interest

The authors declare no conflicts of interest.

## Supporting information


**Figure S1** Construction of TLR3 interference and overexpression vectors. (a) Verification of the Transfection efficiency of VSC4.1 cells. (b) Mean fluorescence intensity of transfected VSC4.l cells (*n* = 3). (c) Quantitative PCR amplification and melting curve analysis assessed TLR3 interference efficacy. (d) Relative expression levels were determined by QPCR after TLR3 interference (*n* = 3). (e) PCR electrophoresis results demonstrate the digestion of AgeI and EcoRI on FC‐023. (f) PCR electrophoretic map of transformed receptor cell DH5α. (g) Vector map representation of FV‐023‐Rat‐TLR3‐shRNA construct. (h) PCR electrophoresis results using Not I /BamH I restriction enzymes on the construct. (i) PCR electrophoresis results were obtained using receptive cells as the template DNA source. (j) Sequencing results confirmed the sequencing accuracy of pcDNA3.1‐Hygro (+)‐Rat‐TLR3. (k) The vector map represents the construction of pcDNA3.1‐Hygro (+)‐Rat‐TLR3. (l) Western blot analysis demonstrates the successful construction of the TLR3 interference (si‐TLR3) and overexpression (oe‐TLR3) vectors. (m) The expression level of TLR3 in panel l was quantified (*n* = 3). Compared with NC, *p < 0.05, **p < 0.01, ***p < 0.005. Compared with NC‐Plasmid (oe), ##*p* < 0.01, ###*p* < 0.005; Compared with oe‐TLR3, ▲▲▲*p* < 0.005; Compared with NC‐Plasmid (si), ♦♦♦*p* < 0.005
**Figure S2** (a, b) Expression of cleaved caspase‐3 at different time points after treatment under 0.1Mpa pressure (normal atmospheric pressure). (c, d) Expression of MAP2 at different time points after treatment under 0.1Mpa pressure (normal atmospheric pressure). (e–g) the correlation between TLR3 and cell apoptosis and caspase‐3. (h) the rsRNA increased in VSC4.1 cells treated with 1.5 MPa pressure (Compared with NC, *p* < 0.005).

## Data Availability

Anonymised data not published within this article will be made available by request from any qualified investigator.
