# Supplementary figures and images for: TLR3 Knockdown Attenuates Pressure‐Induced Neuronal Damage In Vitro
Source: J Cell Mol Med. 2024 Dec 13;28(23):e70276. doi: 10.1111/jcmm.70276 (PMC11640903; doi:10.1111/jcmm.70276)

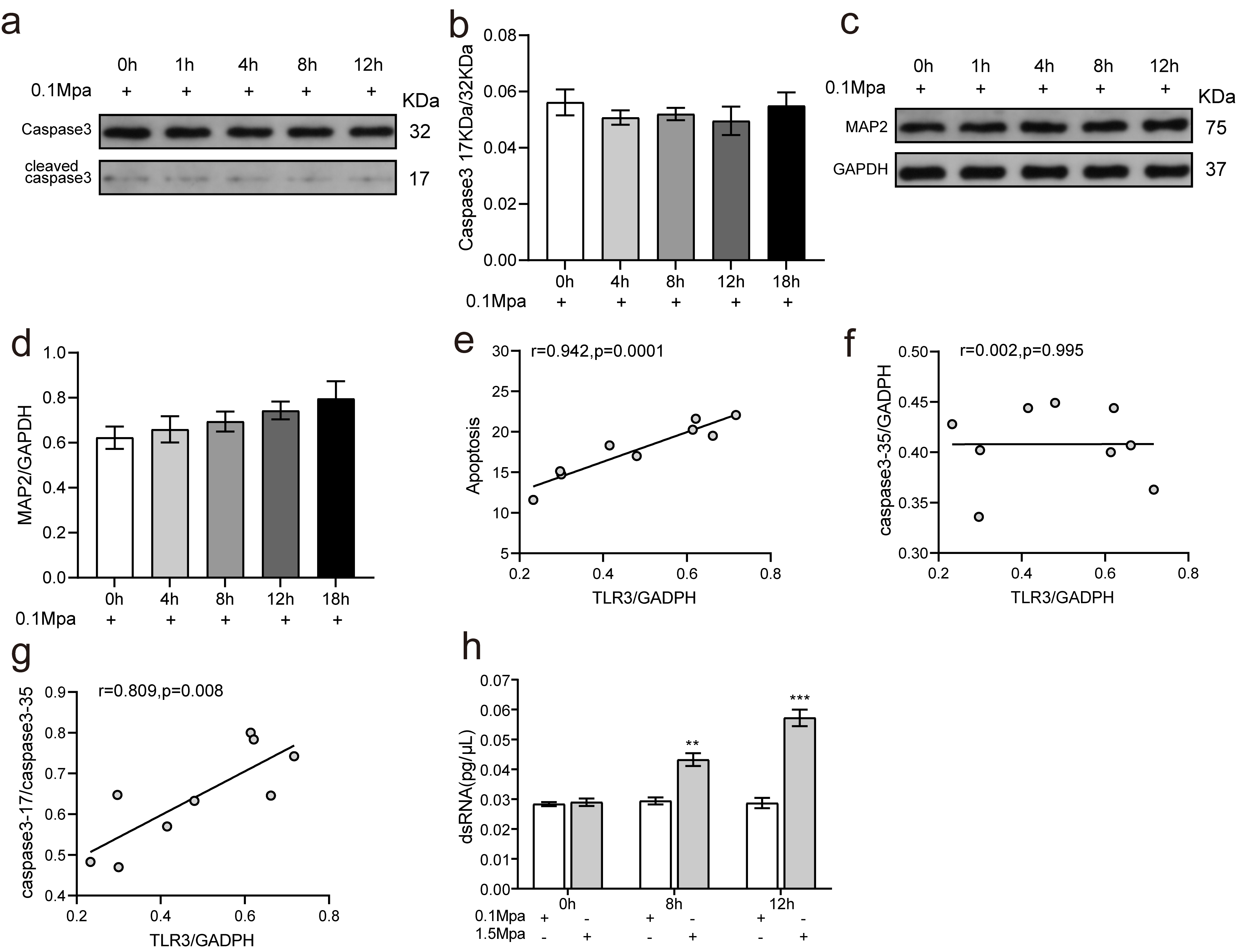

Supplement: Supplementary file 1 — Figure S1 Construction of TLR3 interference and overexpression vectors. (a) Verification of the Transfection efficiency of VSC4.1 cells. (b) Mean fluorescence intensity of transfected VSC4.l cells (n = 3). (c) Quantitative PCR amplification and melting curve analysis assessed TLR3 interference efficacy. (d) Relative expression levels were determined by QPCR after TLR3 interference (n = 3). (e) PCR electrophoresis results demonstrate the digestion of AgeI and EcoRI on FC‐023. (f) PCR electrophoretic map of transformed receptor cell DH5α. (g) Vector map representation of FV‐023‐Rat‐TLR3‐shRNA construct. (h) PCR electrophoresis results using Not I /BamH I restriction enzymes on the construct. (i) PCR electrophoresis results were obtained using receptive cells as the template DNA source. (j) Sequencing results confirmed the sequencing accuracy of pcDNA3.1‐Hygro (+)‐Rat‐TLR3. (k) The vector map represents the construction of pcDNA3.1‐Hygro (+)‐Rat‐TLR3. (l) Western blot analysis demonstrates the successful construction of the TLR3 interference (si‐TLR3) and overexpression (oe‐TLR3) vectors. (m) The expression level of TLR3 in panel l was quantified (n = 3). Compared with NC, *p < 0.05, **p < 0.01, ***p < 0.005. Compared with NC‐Plasmid (oe), ##p < 0.01, ###p < 0.005; Compared with oe‐TLR3, ▲▲▲p < 0.005; Compared with NC‐Plasmid (si), ♦♦♦p < 0.005 Figure S2 (a, b) Expression of cleaved caspase‐3 at different time points after treatment under 0.1Mpa pressure (normal atmospheric pressure). (c, d) Expression of MAP2 at different time points after treatment under 0.1Mpa pressure (normal atmospheric pressure). (e–g) the correlation between TLR3 and cell apoptosis and caspase‐3. (h) the rsRNA increased in VSC4.1 cells treated with 1.5 MPa pressure (Compared with NC, p < 0.005). [file JCMM-28-e70276-s001.zip › jcmm70276-sup-0001-FiguresS1-S2/supplement Fig.2.tif]

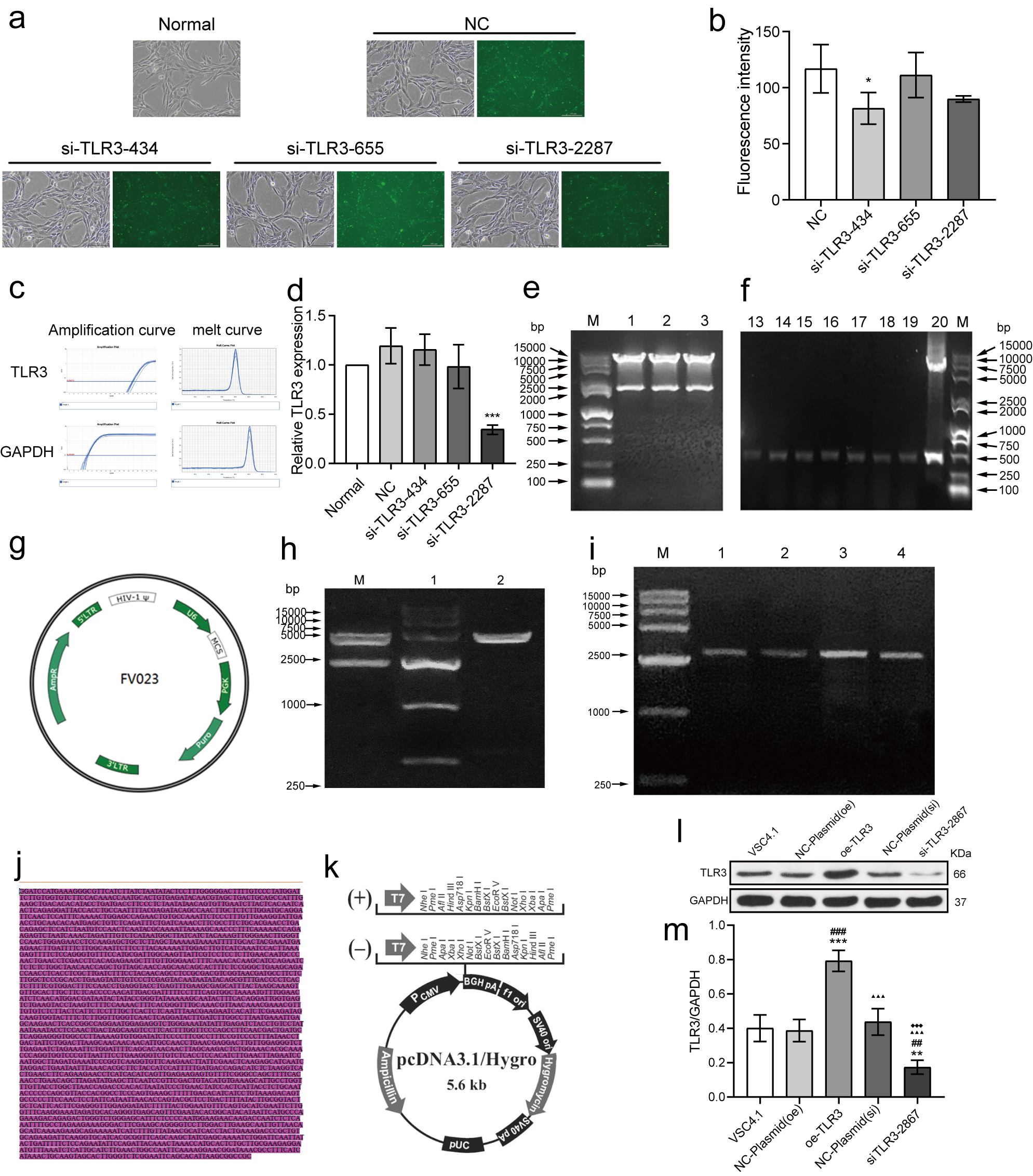

Supplement: Supplementary file 1 — Figure S1 Construction of TLR3 interference and overexpression vectors. (a) Verification of the Transfection efficiency of VSC4.1 cells. (b) Mean fluorescence intensity of transfected VSC4.l cells (n = 3). (c) Quantitative PCR amplification and melting curve analysis assessed TLR3 interference efficacy. (d) Relative expression levels were determined by QPCR after TLR3 interference (n = 3). (e) PCR electrophoresis results demonstrate the digestion of AgeI and EcoRI on FC‐023. (f) PCR electrophoretic map of transformed receptor cell DH5α. (g) Vector map representation of FV‐023‐Rat‐TLR3‐shRNA construct. (h) PCR electrophoresis results using Not I /BamH I restriction enzymes on the construct. (i) PCR electrophoresis results were obtained using receptive cells as the template DNA source. (j) Sequencing results confirmed the sequencing accuracy of pcDNA3.1‐Hygro (+)‐Rat‐TLR3. (k) The vector map represents the construction of pcDNA3.1‐Hygro (+)‐Rat‐TLR3. (l) Western blot analysis demonstrates the successful construction of the TLR3 interference (si‐TLR3) and overexpression (oe‐TLR3) vectors. (m) The expression level of TLR3 in panel l was quantified (n = 3). Compared with NC, *p < 0.05, **p < 0.01, ***p < 0.005. Compared with NC‐Plasmid (oe), ##p < 0.01, ###p < 0.005; Compared with oe‐TLR3, ▲▲▲p < 0.005; Compared with NC‐Plasmid (si), ♦♦♦p < 0.005 Figure S2 (a, b) Expression of cleaved caspase‐3 at different time points after treatment under 0.1Mpa pressure (normal atmospheric pressure). (c, d) Expression of MAP2 at different time points after treatment under 0.1Mpa pressure (normal atmospheric pressure). (e–g) the correlation between TLR3 and cell apoptosis and caspase‐3. (h) the rsRNA increased in VSC4.1 cells treated with 1.5 MPa pressure (Compared with NC, p < 0.005). [file JCMM-28-e70276-s001.zip › jcmm70276-sup-0001-FiguresS1-S2/Supplementary Fig. 1.tif]
